# A plasmid-encoded detoxification system contributing to the survival of *Klebsiella oxytoca* complex bacteria in the presence of the isothiazolinone biocide MIT/CMIT

**DOI:** 10.1128/aem.01970-25

**Published:** 2026-05-29

**Authors:** Samuel Hauf, Ralf Dieckmann, Sascha Al Dahouk, Szilvia Neuhaus

**Affiliations:** 1 German Federal Institute for Risk Assessment 27652, Berlin, Germany; 2 Department 1 - Infectious Diseases, Robert Koch Institute 9222, Berlin, Germany; Washington University in St Louis, St. Louis, Missouri, USA

**Keywords:** mechanisms of resistance, glutathione, co-resistance

## Abstract

Biocides are widely used to control unwanted biological growth. Isothiazolinone biocides are one class of biocides used to stabilize a range of products against microbial contamination and degradation. Although the development of resistance to such biocides is thought to be unlikely, we describe a system capable of detoxifying the common isothiazolinone biocide MIT/CMIT (2-methyl-4-isothiazolin-3-one and 5-chloro-2-methyl-4-isothiazolin-3-one). It was found in *Klebsiella oxytoca* complex bacteria contaminating a commercial liquid soap that contained the isothiazolinone as a preservative. The isolate inactivates MIT/CMIT via a glutathione-dependent reaction catalyzed by a plasmid-encoded glutathione transferase system. This system comprises a glutathione transferase (*gstA*) and a hydroxyacylglutathione hydrolase (*gloB*), both located in one operon on an 80-kbp plasmid and co-localized with an arsenic resistance cassette. The plasmid can be transferred by conjugation to *Escherichia coli*. The findings highlight the risk that broadening biocide use can increasingly drive the evolution, selection, and spread of different resistance mechanisms.

**IMPORTANCE:**

Biocide usage is increasing worldwide to combat organisms causing spoilage or infectious disease. However, the use of biocides is also recognized as a selection factor that shapes microbial communities and drives adaptation. Isothiazolinone biocides are mainly used to stabilize liquid products, personal care products, and fuel against microbial spoilage. The detoxification system described here allowed bacteria to survive in a liquid soap cleaning product containing the widely used biocide preservative MIT/CMIT. Importantly, the biocide detoxification system is mobile between different species and co-localized with a metal resistance operon. The system thereby demonstrates how the spread and co-selection of resistances can occur in bacterial populations exposed to biocides.

## INTRODUCTION

Modern society utilizes different chemicals to perform specialized functions. According to common definitions (e.g., that of the European Union), biocidal products are used “… with the intention of destroying, deterring, rendering harmless, preventing the action of, or otherwise exerting a controlling effect on, any harmful organism.” (1). Harmful organisms can include pests, such as insects or rats, and microorganisms, such as bacteria. Disinfectants are generally used to kill microorganisms, while preservatives are added to products to stabilize them against microbial spoilage. Preservatives are widely used in foods (e.g., nitrites, sulfites, sorbates) and consumer or personal care products (e.g., benzoate, triclosan, parabens), as well as in *in vitro* diagnostics (e.g., azide, thimerosal).

A widely used preservative is the combination of 2-methyl-4-isothiazolin-3-one (MIT) and 5-chloro-2-methyl-4-isothiazolin-3-one (CMIT). MIT/CMIT is used to stabilize personal care products, fuel (marketed as Kathon), and *in vitro* diagnostics (marketed as ProClin) against microbial spoilage. In the European Union, the United States of America, and Canada, MIT/CMIT can be added to consumer products, such as soaps and shampoos, at a maximum concentration of 15 ppm (15 mg/L) as of 2025 (2–4). As a fuel preservative, it is used at 100 ppm (5).

Although concerns regarding the adverse effects of biocides like MIT/CMIT, associated with their ongoing large-scale use, have been reported (6–8), uncertainties remain regarding their environmental risks. The widespread use of biocides can promote the selection and dissemination of resistance determinants within exposed bacteria populations (9, 10). Of particular relevance is the co-localization of multiple resistance genes on mobile genetic elements, such as plasmids (11, 12), where exposure to one compound can co-select for additional resistances (12–14). In the context of biocides, the terms resistance and tolerance are not uniformly defined (15–17). In this study, resistance refers to a genetically encoded reproducible increase in the minimal inhibitory concentration (MIC), defined as the lowest concentration of a biocide that inhibits bacterial growth. While the minimal bactericidal concentration (MBC) may additionally be used to assess bactericidal activity, the present study focuses on MIC-based susceptibility changes.

Resistances to antimicrobial substances are usually caused by one of the following mechanisms or a combination thereof: (i) reduced intracellular accumulation due to increased efflux or decreased membrane permeability, (ii) modification of the target site, resulting in reduced sensitivity, or (iii) enzymatic inactivation/detoxification of the antimicrobial, leading to its inactivation (18–20). The active extrusion of biocides, mediated by efflux pumps, is a common method of resistance in bacteria (21, 22). Broad-specificity pumps, including members of RND family, such as AcrAB-TolC, can export structurally diverse compounds (23, 24). Examples like the mentioned AcrAB-TolC system are multi-drug exporters that can target antibiotics, toxic elements, or biocides (24–27). Another resistance mechanism is the modification/detoxification of the biocide. In this case, specialized enzymes can cleave or modify biocidal compounds, leading to a loss of activity. This is the case for peroxide-decomposing enzymes (28). Radical defense systems can also mediate resistance against different electrophilic biocides like MIT/CMIT (16).

The biocide MIT/CMIT is marketed as “effectively and immediately inhibiting a broad spectrum of microbes” by interfering with four separate enzymes in the Krebs cycle (pyruvate dehydrogenase, α-ketoglutarate dehydrogenase, succinate dehydrogenase, and NADH dehydrogenase) (29). Its antimicrobial activity is primarily attributed to its high reactivity with thiol groups of such essential enzymes (30). In line with this multi-target mode of action, most bacterial species exhibit low MICs, commonly below 1 mg/L, and are therefore considered susceptible (16). Although MIC values above 10 mg/L have been reported for certain organisms, no specific, well-characterized resistance mechanism has been conclusively demonstrated to account for the reduced susceptibility (16, 31). Based on such observations, the development of resistance has been considered unlikely (ProClin description on https://www.sigmaaldrich.com). However, in 2013 routine surveillance detected a liquid soap containing the biocide MIT/CMIT as a preservative contaminated with bacteria at more than 10^4^ colony-forming units/mL (CFU/mL). The causative agent was a species of *Klebsiella oxytoca* complex bacteria (32, 33). Bacteria belonging to the *Klebsiella oxytoca* complex are opportunistic pathogens known for their ability to carry, acquire, and disseminate multiple virulence genes or resistance determinants (34). Isolates recovered from the product had elevated MIC values (2–8 mg/L) that were higher than those generally reported for susceptible bacteria (1 mg/L) indicating reduced susceptibility to MIT/CMIT. We investigated the molecular basis underlying this phenotype with the objective of identifying the genetic determinant responsible for MIT/CMIT detoxification and assessing its potential mobility.

## MATERIALS AND METHODS

### Chemicals

N-laurylsarcosine (L9150), MIT/CMIT (ProClin 300; 48912-U), and benzisothiazolinone (BIT; 561487) were obtained from Sigma. Potassium antimony(III) oxide tartrate (1H4E.1), streptomycin (HP66.1), ampicillin (K029.2), acetic acid (3738.1), ethanol (5054.1), Tris-ethylenediaminetetraacetic acid (Tris-EDTA or TE) buffer (1052.1), and EDTA (8040.1) were obtained from Carl Roth. Agarose for gel electrophoresis was obtained from Invitrogen (16500-500). Tryptic soy agar (1.05458), LB (Luria-Bertani) broth (1.10285), glycerol (104094), sodium chloride (1.06404), and sodium hydroxide (1.06498) were obtained from Merck. Sodium dodecyl sulfate (SDS) (A15020250) was obtained from AppliChem. The Eugon medium (AEB610573) was obtained from Biomerieux. Proteinase K (03115828001) was obtained from ROCHE.

### Strains and culture conditions

All bacteria (see Table 1) were grown at 37°C on tryptic soy agar or in LB broth under aseptic conditions with shaking at 150 rpm, unless otherwise indicated. Liquid cultures were grown in glass tubes with 2–3 mL of the medium capped with cellulose. Cells were stored at −80°C in the presence of 20% (v/v) glycerol.

**TABLE 1:** Strains used in this study

Strain	Species	Description
PHS-890^*a*^	*Klebsiella oxytoca* complex	Soap isolate, bottle 1
PHS-891^*a*^	*Klebsiella oxytoca* complex	Soap isolate, bottle 1
PHS-892^*a*^	*Klebsiella oxytoca* complex	Soap isolate, bottle 1
PHS-893^*a*^	*Klebsiella oxytoca* complex	Soap isolate, bottle 1
PHS-894^*a*^	*Klebsiella oxytoca* complex	Soap isolate, bottle 1
PHS-895^*a*^	*Klebsiella oxytoca* complex	Soap isolate, bottle 2
PHS-896^*a*^	*Klebsiella oxytoca* complex	Soap isolate, bottle 2
PHS-897^*a*^	*Klebsiella oxytoca* complex	Soap isolate, bottle 2
PHS-898^*a*^	*Klebsiella oxytoca* complex	Soap isolate, bottle 2
PHS-899^*a*^	*Klebsiella oxytoca* complex	Soap isolate, bottle 2
ATCC13182	*Klebsiella oxytoca*	Reference strain
DSM12082	*Klebsiella pneumoniae*	Reference strain
MG1655	*Escherichia coli*	Reference strain
TOP10	*Escherichia coli*	Cloning strain (C303003, Thermo)
10*β*	*Escherichia coli*	Cloning strain (C3019I, NEB)
BW25113	*Escherichia coli*	KEIO collection; parental strain
JW2663	*Escherichia coli*	KEIO collection; Δ*gshA*

^*a*^ The soap isolates were obtained from two lots (two different bottles) of the same contaminated soap batch (32).

### Oligonucleotides

Oligonucleotide primers used in this study are listed in Table 2.

**TABLE 2:** Oligonucleotide primers used in this study^*a*^

Oligo name	Sequence 5′−3′
BfRSaH001	gactctagaggatccccagcccgcactaagca
BfRSaH003	tcgagctcggtacccgttcaccggtgaatctgc
BfRSaH018	gactctagaggatccccactggcgcataaccatcc
BfRSaH022	gactctagaggatccccctcagcaagagcatcagt
BfRSaH024	tcgagctcggtaccccagacatggaagtgcaca

^*a*^ All oligos were ordered desalted from metabion international AG, Germany.

### MIC determination

Liquid cultures were grown overnight (18 ± 2 h) and then diluted to around $10^{6}$ CFU/mL by adjusting the optical density (OD) at 595 nm to 0.1 using a 1 cm polystyrene cuvette (634-0675, VWR) in a cell density meter (model 40, Fisher Scientific) blanked against uninoculated medium. Next, 150 μL of the inoculum was added to 50 μL of the medium containing four times the intended concentration (final concentrations: 0, 0.25, 0.5, 1, 2, 4, 8, or 16 mg/L) of MIT/CMIT in 96-well plates (10861-562, Corning). The plates were incubated statically overnight (18 ± 2 h) at 37°C. Afterwards, the OD at 595 nm was measured (Biorad iMark microplate reader). The MIC was determined as the lowest concentration of MIT/CMIT at which no increase in the OD (compared with sterile medium control) was detected (35). Three independent experiments (biological replicates) were performed. The values presented are averages ± standard deviations.

### Plasmid profile analysis

Plasmids smaller than 80 kbp were purified using the QIAprep Spin Miniprep Kit (Qiagen, 27104) following the manufacturer’s protocol for lysis and neutralization. Briefly, 4 mL of an overnight culture (18 ± 2 h) was subjected to alkaline lysis using buffer P2 (containing sodium hydroxide and SDS), followed by neutralization with buffer N3. After centrifugation at 18,000 × *g* for 10 min, the supernatant containing the plasmids was precipitated with three volumes of absolute ethanol, resuspended in molecular biology grade water, run on a standard 0.8% (w/v) agarose gel, and stained with peqGREEN (Avantor, 732-3196) for visualization under UV illumination.

For the visualization of plasmids bigger than 80 kbp, cells from an agar plate were resuspended in TE buffer (10 mM Tris-HCl, 1 mM EDTA, pH 8.0), and the OD at 595 nm was adjusted to 1.0. The suspension (0.4 mL) was supplemented with 20 μL of a 20 mg/mL proteinase K solution and then mixed with 0.4 mL of a 2% (w/v) SeaKem Gold-Agarose (50150, Lonza) and 1% (w/v) SDS solution. Agarose containing bacteria was left to solidify in CHEF Disposable Plug Molds (1703713, BIO-RAD) for 30 min. After solidification, each plug was transferred into 5 mL cell lysis buffer (50 mM Tris-HCl pH 8.0, 50 mM EDTA, 1% (v/v) N-laurylsarcosine, 0.1 mg/mL proteinase K) for 2 h at 54°C with shaking at 150 rpm. They were then washed twice at 50°C in 15 mL of pure water (prewarmed to 50°C), followed by four times with 15 mL TE buffer (prewarmed to 50°C). Blocks were then transferred into 1% (w/v) SeaKem Gold-Agarose gels. Pulsed-field gel electrophoresis (CHEF DR III, Bio-Rad) was run at 6 V/cm and 120°C, with an initial switch time of 2.2 s and a final switch time of 54.2 s for 20 h at 14°C. H9812 standard (*Salmonella enterica*, serotype Braenderup H9812) was used as a size reference. Gels were stained with ethidium bromide and visualized under UV illumination.

### PacBio sequencing and data availability

The soap isolate PHS-894 (containing the 80-kbp plasmid) was submitted to GATC Biotech AG (Germany) for PacBio RS sequencing in 2016, yielding a total of 127,062 sequencing reads with 1,276,475,000 bases, corresponding to an approximate sequencing depth of 200-fold. The processed read data for the plasmid are available at ENA (European Nucleotide Archive project accession: PRJEB89844). An annotated file in Genebank format is provided as supplementary material.

### Survival tests in soap and LB

A total of 25 mL of liquid soap (same brand as the contaminated product with MIT/CMIT as the preservative) was added to an Erlenmeyer flask. The soap was inoculated with 10^5^ CFU/mL (through the addition of 200 μL of an overnight culture at approximately 10^8^ CFU/mL (estimated based on OD measurement) and mixed carefully using a magnetic stirrer for 5 min. The mix was incubated in the dark at room temperature for the indicated time (up to 22 days). At each time point, 1 mL of the soap was added to 9 mL of Eugon medium to neutralize the preservative for 15 min at room temperature. This solution was diluted in duplicate from 10^0^ to 10^−4^ in 0.9% (w/v) NaCl. Next, 100 μL of each dilution was plated on tryptic soy agar plates and incubated overnight to determine the number of colonies. The number of colony-forming units per mL was calculated (CFU/mL). Experiments were performed in triplicate and the averages ± standard deviations are reported. Survival in the presence of MIT/CMIT was tested in a manner similar to the soap testing, but using LB medium containing 1.9, 3.8, or 7.5 mg/L of MIT/CMIT, prepared by dilution of a freshly made 3,000 mg/L aqueous stock solution.

### Conjugation

Liquid cultures of soap isolate PHS-894 [MIT${}^{R}$] and TOP10 (*rpsL* [Str${}^{R}$]) were grown overnight in LB and subsequently diluted in fresh medium to an OD at 595 nm of 0.5. Next, 100 μL of the donor (PHS-894) was mixed with 200 μL of the recipient (TOP10) and incubated statically at 37°C for 3 h. Afterwards, 100 μL of a 1:100 dilution in fresh LB medium was plated on LB agar containing 25 µg/mL streptomycin and 0.45 mg/L MIT/CMIT. Small colonies that appeared after 18 h at 37°C were streaked on new plates containing both streptomycin and MIT/CMIT at the same concentrations and incubated for 18 ± 2 h. Single colonies were tested for the presence of the 80-kbp plasmid by polymerase chain reaction (PCR) using primers BfRSaH001 and BfRSaH003 (see Table 2). Two transconjugants were grown in liquid medium containing 25 µg/mL streptomycin and 0.45 mg/L MIT/CMIT. Glycerol stocks were made from these cultures after overnight incubation (18 ± 2 h). The strains were grown from these stocks on plates without streptomycin and MIT/CMIT for all subsequent experiments.

### Cloning

The 80-kbp plasmid was purified from strain PHS-894 using the QIAprep Spin Miniprep Kit (Qiagen, 27104) following the manufacturer’s protocol. The arsenic resistance operon *arsAB* and surrounding regions (5,852 bp) were amplified from purified 80-kbp plasmid with primers BfRSaH003 and BfRSaH018 (see Table 2) using the TaKaRa Ex Premier DNA Polymerase Master Mix. The resulting fragment was digested with BamHI-HF (R3136L, NEB) and KpnI-HF (R3142L, NEB). The pUC19 vector (SD0061, Thermo) was cut using the same enzymes, with the addition of Quick CIP (M0525L, NEB) for dephosphorylation. The digested fragments were purified using DNA Clean and Concentrator-5 (D4004, Zymo) and ligated using the Quick Ligation Kit (M2200L, NEB). Next, 3 μL of the ligation was transformed into 50 μL of chemically competent 10*β* cells (C3019I, NEB) through heat shock at 42°C for 45 s. Transformants were selected on LB plates with ampicillin at 100 μg/mL. The glutathione operon *gstA-gloB* and surrounding regions, including the native promoter (2,126 bp), was also amplified from purified 80-kbp plasmid using primers BfRSaH022 and BfRSaH024 (see Table 2). It was cloned as described for the arsenic resistance operon *arsAB*. Plasmid sequences were confirmed by whole plasmid sequencing (Oxford Nanopore sequencing performed by Genewiz, average sequencing depth between 1,000- and 2,000-fold) and are available at ENA (project accession: PRJEB89844).

### Agar diffusion tests

An overnight culture of the strain to be tested was cross-streaked in three directions onto agar plates using sterile cotton buds (EH11.1, Carl Roth) soaked in the culture. Immediately after the streaking of the culture, 5 μL of the respective stock solution was directly spotted onto the agar surface. For MIT/CMIT, the solution spotted was at 3,000 mg/L, while benzisothiazolinone (BIT) was 9,000 mg/L, and potassium antimony(III) oxide tartrate was used at 50 mM. These concentrations were selected to generate measurable inhibition zones under the assay conditions while allowing discrimination between strains differing in the respective detoxification genes. The plates were incubated at 37°C overnight before the diameter of the inhibition zone was measured using a ruler. Pictures of the plates were taken with a Canon IXUS 185 digital camera under identical lighting condition with a ring light. Brightness and contrast were uniformly adjusted for all images solely for visualization purposes. Agar diffusion tests were repeated at least three times independently.

## RESULTS

### Isolates with reduced MIT/CMIT susceptibility all contain an 80-kbp plasmid

Local government agencies obtained different *K. oxytoca* complex isolates from two bottles of the same batch of a liquid soap product that contained MIT/CMIT as a preservative (32, 33). Detailed information on the complete chemical composition of the commercial soap product was not available. According to the product label, MIT/CMIT was listed as preservative. However, products containing MIT/CMIT that might come into contact with unprotected skin typically contain the preservative at concentrations ranging from 1 to 15 mg/L (36). In total, 6 out of 10 isolates showed fourfold higher MIC values (8 mg/L) than the remaining isolates (1.7–2 mg/L) (Table 3) and also higher MIC values than the reference strains (Table 4).

**TABLE 3:** MIC and plasmid profile of the *Klebsiella* isolates from soap

Strain number	MIC^*a*^ [mg/L]	Plasmids^*b*^				
		160 kbp	80 kbp	18 kbp	16 kbp	4 kbp
PHS-890	2 ± 0	+	−	+	−	+
PHS-891^*c*^	8 ± 0	+	+	−	−	+
PHS-892	2 ± 0	+	−	+	−	+
PHS-893^*c*^	8 ± 0	+	+	−	−	+
PHS-894^*c*^	8 ± 0	+	+	−	−	+
PHS-895	1.7 ± 0.6	+	−	+	−	+
PHS-896	1.7 ± 0.6	+	−	−	+	+
PHS-897^*c*^	8 ± 0	+	+	−	−	+
PHS-898^*c*^	8 ± 0	+	+	−	−	+
PHS-899^*c*^	8 ± 0	+	+	−	−	+

^*a*^ The MIC was determined by LB broth microdilution. Three independent experiments were performed and the values presented are averages ± standard deviations.

^*b*^ The presence of plasmids and their approximate sizes are based on the results of plasmid profile analysis.

^*c*^ The gray shading highlights all isolates with an increased MIC.

**TABLE 4:** MIC values of MIT/CMIT for selected reference strains and *E. coli* transconjugants^*a*^

Strain	Species	MIC [mg/L]
ATCC13182	*K*. *oxytoca*	2 ± 0
DSM 12082	K. *pneumoniae*	2 ± 0
MG1655	*E*. *coli*	2 ± 0
TOP10	*E*. *coli*	1 ± 0
PHS-894 - > TOP10 transconjugant 1	*E*. *coli*	5.3 ± 2.3
PHS-894 - > TOP10 transconjugant 2	*E. coli*	4 ± 0

^*a*^ MIC values were determined by LB broth microdilution. Values represent the mean ± standard deviation of three independent experiments.

As previously reported, whole-genome comparison revealed a high degree of chromosomal relatedness among these isolates, but additional bands in PFGE indicated the existence of plasmids in some isolates (33). Indeed, plasmid profile analysis, based on plasmid size determination by agarose gel electrophoresis, revealed distinct plasmid patterns among the isolates, which were subsequently evaluated for their association with MIC values (Table 3). An approximately 80-kbp plasmid was present in all isolates with MIC values of 8 mg/L but absent in all strains with MIC values around 2 mg/L (Table 3). The association of an increased MIC with the occurrence of the 80-kbp plasmid indicates the presence of resistance determinants located on this plasmid.

**Fig 1:**
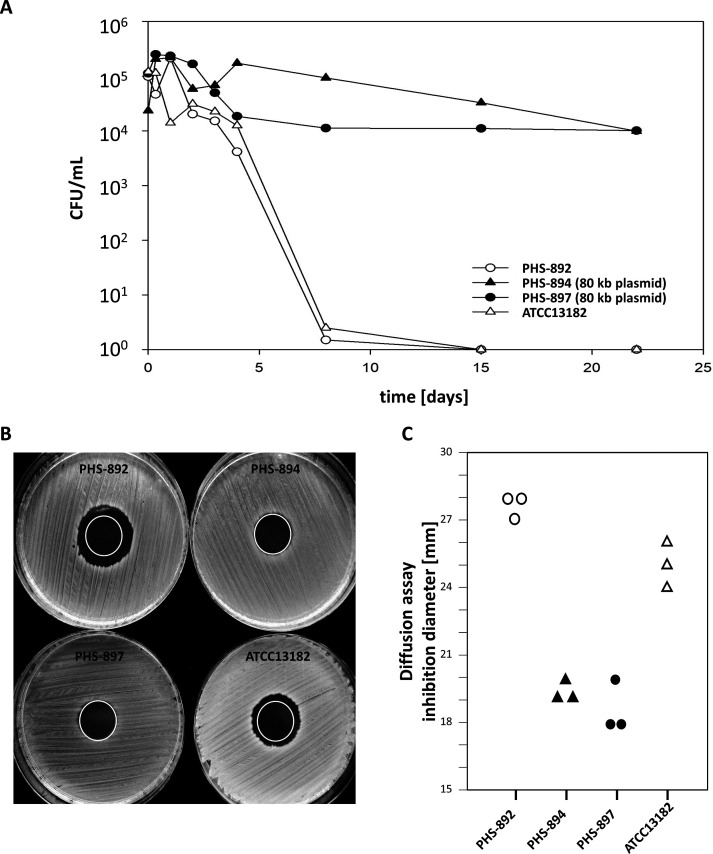
Survival of representative isolates in the presence of MIT/CMIT. (**A**) Survival in soap with MIT/CMIT determined as CFU/mL over time demonstrated that isolates PHS-894 and PHS-897 (MIT/CMIT MIC = 8 mg/L) survived in the original soap brand for more than 20 days at room temperature (black symbols). In contrast, isolate PHS-892 (MIT/CMIT MIC = 2 mg/L) and the reference strain ATCC13182 were completely inactivated within 15 days (white symbols). (**B**) Representive image of the agar diffusion assay. MIT/CMIT (5 μL of a 3,000 mg/L MIT/CMIT solution) placed on a lawn of bacteria produced a bigger zone without growth in susceptible *Klebsiella* strains (PHS-892 and ATCC13182) and a smaller inhibition zone in less susceptible strains (PHS-894 and PHS-897). The white ring indicates the boundary of the inhibition zone for isolate PHS-894 (MIT/CMIT MIC = 8 mg/L). (**C**) Diameter of the inhibition zone from diffusion assays of three independent experiments.

The elevated MIC of 8 mg/L observed for isolates PHS-891, PHS-893, PHS-894, PHS-897, PHS-898, and PHS-899, compared with the approximately fourfold lower MIC of isolates PHS-890, PHS-892, PHS-895, and PHS-896 (Table 3), might contribute to survival inside the soap containing MIT/CMIT. To test this hypothesis, a fresh (uncontaminated) batch of the same soap brand was spiked with representative isolates, namely PHS-892, PHS-894, and PHS-897, and the reference strain ATCC13182 at 10^5^ CFU/mL. The isolates with MIC values of 8 mg/L survived inside the soap for more than 20 days, exhibiting 1 log_${}_{10}$_ reduction in CFU/mL (from 10^5^ to 10^4^ CFU/mL) over this period. In contrast, the isolate PHS-892 and the reference strain ATCC13182 (with MIT/CMIT MICs of 2 mg/L) died within 15 days (Fig. 1A). Isolates with reduced susceptibility to MIT/CMIT (PHS-894 and PHS-897 with MIC values of 8 mg/L) could also grow at higher concentrations of MIT/CMIT on agar, as shown by the smaller zone of growth inhibition in the agar diffusion assay (Fig. 1B and C).

Strain ATCC13182 showed inhibition zone diameters comparable to those of the susceptible soap isolate PHS-892 (MIT/CMIT MIC = 2 mg/L) and clearly larger than those observed for the isolates with reduced susceptibility (MIT/CMIT MIC = 8 mg/L) (Fig. 1C). Specifically, PHS-892 exhibited inibition zones of 27–28 mm, whereas ATCC13182 showed slightly smaller values of 24–26 mm. In contrast, the isolates with reduced susceptiblity (PHS-894 and PHS-897) showed substantially smaller inhibition zones of 18–20 mm. Consistent with this observation, the MIC of ATCC13182 was identical to that of the susceptible isolates (2 mg/L, see Table 4).

**Fig 2:**
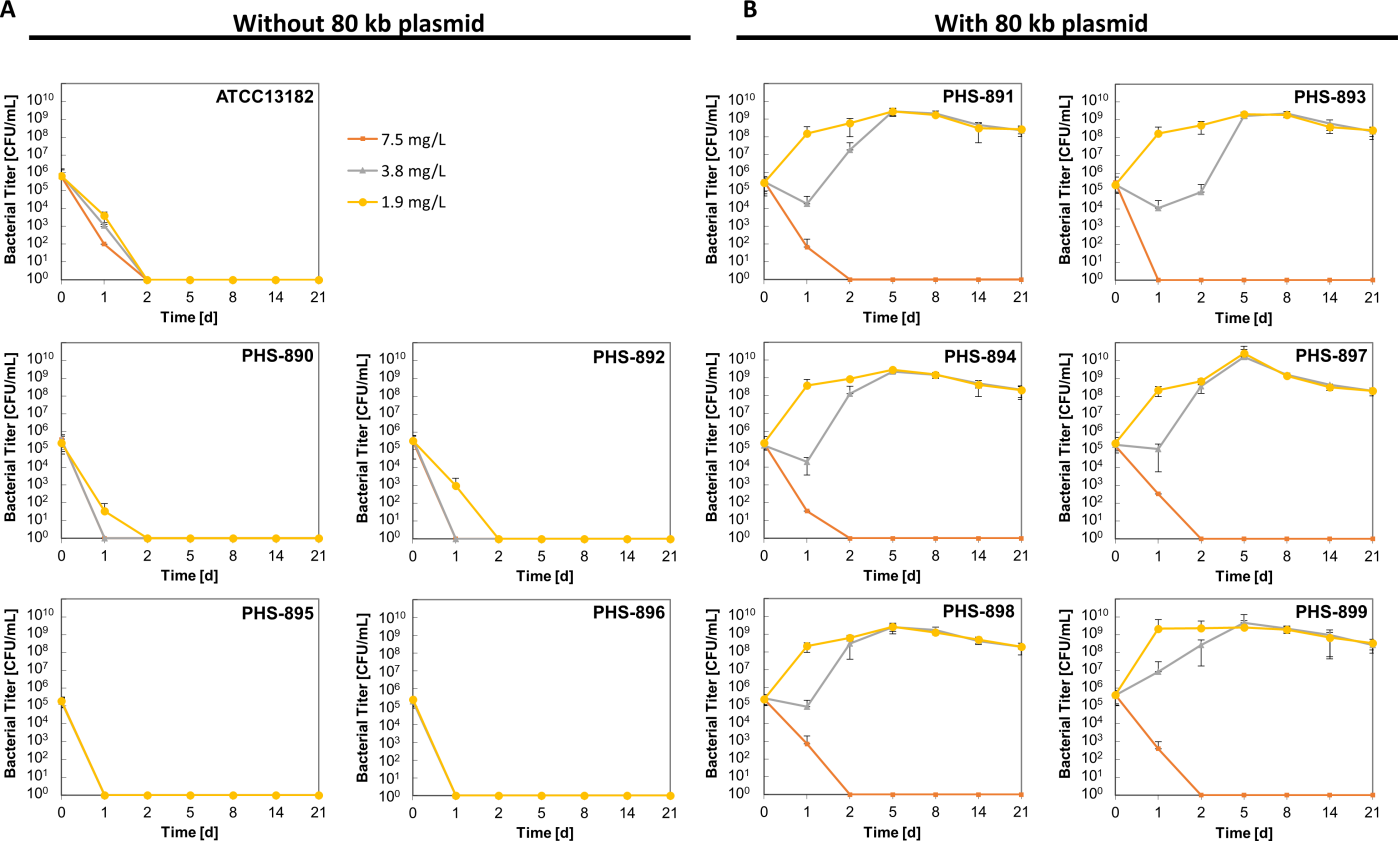
Survival kinetics of *Klebsiella oxytoca* complex soap isolates (PHS-890 to PHS-899) and reference strain ATCC13182 in LB broth containing defined MIT/CMIT concentrations. The number of colony-forming units per mL of LB broth over 21 days at room temperature in the presence of MIT/CMIT (7.5 mg/L, orange; 3.8 mg/L, gray; 1.9 mg/L, yellow) is presented. Experiments were performed in triplicate, and the averages ± standard deviations are shown. (**A**) MIT/CMIT readily killed all susceptible isolates at 1.9 mg/L. (**B**) Isolates with a higher MIC of 8 mg/L could survive and grow in the presence of up to 3.8 mg/L MIT/CMIT (yellow and gray curves).

The soap isolates were also grown in LB broth containing MIT/CMIT at concentrations of 1.9, 3.8, and 7.5 mg/L to determine survival kinetics under controlled conditions. The biocide was added to the medium prior to inoculation. The less susceptible isolates (MIC = 8 mg/L) could grow in the presence of up to 3.8 mg/L MIT/CMIT (Fig. 2B), while the strains with a lower MIC (2 mg/L) died within 2 days at 1.9 mg/L (Fig. 2A). At 7.5 mg/L, all strains died within 2 days (Fig. 2). Interestingly, the killing observed in LB broth (Fig. 2) was much faster than the killing in liquid soap (Fig. 1). In summary, survival in the commercial liquid soap product, the increased MIC, and the growth observed in the presence of up to 3.8 mg/L MIT/CMIT indicate that isolates PHS-891, PHS-893, PHS-894, PHS-897, PHS-898, and PHS-899 carry a resistance determinant.

### Horizontal transfer of the MIT/CMIT resistance determinant to recipient *Escherichia coli*

Next, we investigated whether the resistance determinant is mobile and can spread from *Klebsiella* to another bacterial species by conjugation. Such a transfer of the MIT/CMIT resistance determinant as a result of plasmid mobility would enable rapid spread in populations under selection pressure. Isolate PHS-894, as the plasmid donor, was statically incubated with One Shot TOP10 *E. coli* (C404010, Thermo), which is streptomycin-resistant. After 3 h at 37°C, transconjugants were selected on agar plates containing MIT/CMIT and streptomycin. The MIC of MIT/CMIT was determined for two *E. coli* transconjugants, which showed a fourfold increased MIC compared with the parental strain (Table 4). The increase was similar to that observed in the *K. oxytoca* soap isolates (Table 3).

This result demonstrates that the 80-kbp plasmid and its encoded resistance genes can spread between bacterial species. The exact level of susceptibility to MIT/CMIT is strain-dependent because the MIC for the *E. coli* TOP10 transconjugants is 4 mg/L, while the *K. oxytoca* isolates from soap had an MIC of 8 mg/L. However, TOP10 without the plasmid also has a lower MIC than *K. oxytoca* isolates without the plasmid indicating that additional resistance mechanisms might exist in the *K. oxytoca* isolates (compare Tables 3 and 4).

### Plasmid characterization

To determine the molecular basis of reduced susceptibility to MIT/CMIT, the isolate PHS-894 was sequenced with PacBio. The sequencing data showed that the 80-kbp plasmid is 81,837 bp in size and very similar to pCESC2 (National Center for Biotechnology Information Reference Sequence: NZ_MF156266.1), a plasmid isolated from *E. coli*. Both plasmids contain many transposon-, mobility-, and phage-related genes, but they also contain a putative arsenic resistance operon. This putative arsenic resistance operon includes an ATP-binding-cassette (ABC)-type efflux pump (ArsB). No other known resistance genes were initially identified on the plasmid.

**Fig 3:**
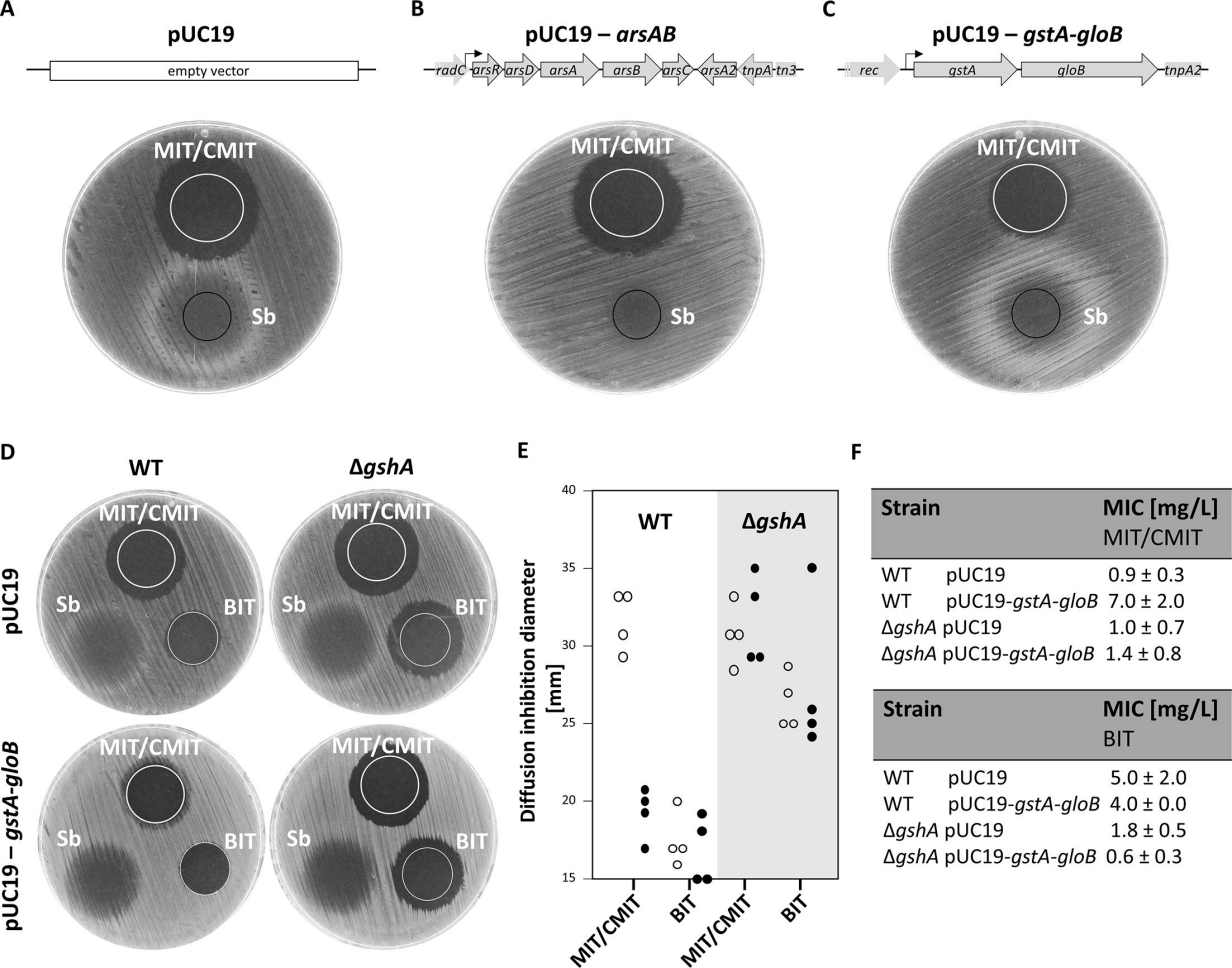
Identification of the genes causing reduced MIT/CMIT susceptibility. Panels **A-C** show the plasmid constructs (top) and the corresponding agar diffusion assay results for the respective strain (bottom). (**A**) *E. coli* 10*β* cells carrying the empty vector pUC19 were sensitive to both MIT/CMIT and antimony (Sb). (**B**) Transfer of the *ars* operon onto pUC19 conferred resistance to antimony but not to MIT/CMIT. (**C**) Transfer of the glutathione operon *gstA-gloB* onto pUC19 reduced susceptibility to MIT/CMIT but not to antimony. (**D**) Effect of the glutathione operon on the susceptibility to MIT/CMIT, BIT, and antimony in diffusion assays. The glutathione operon reduced susceptibility to MIT/CMIT but not to the related biocide BIT (benzisothiazolinone) in *E. coli* K-12 BW25113. In the glutathione-deficient strain (Δ*gshA*), sensitivity to BIT was increased, while the *gstA-gloB* operon did not impact the susceptibility to MIT/CMIT. The susceptibility to antimony was not affected. (**E**) Quantification of the diffusion inhibition zones of MIT/CMIT and BIT in strains with (solid circles) and without (open circles) the glutathione operon *gstA-gloB*. Wild-type strains are shown on a white background and the glutathione-deficient strains on a gray background. (**F**) MIC values of MIT/CMIT and BIT for the WT strain BW25113 and its GshA-deficient mutant presented as the averages ± standard deviations of four independent experiments.

The *ars* operons are known to mediate resistance against arsenic and antimony in *E. coli* via an active efflux mediated by the ArsB pump (37, 38). As a known efflux system, the *ars* operon might also mediate the efflux of MIT/CMIT, which could explain the reduced susceptibility to MIT/CMIT of strains carrying the plasmid. The plasmid-borne *arsB* gene, encoding the actual efflux pump, shows 87.6% sequence identity to the genomic *arsB* of PHS-894. To determine whether this operon contributes to the observed reduction in biocide susceptibility, we first cloned the arsenic resistance cassette from the 80-kbp plasmid into pUC19 and assessed any changes in the susceptibility to antimony and MIT/CMIT in the recipient strain. For this purpose, a 5,852 bp region encompassing the arsenic resistance cassette, as well as surrounding regions (including the promoter), was cloned. Diffusion tests revealed that this region mediated antimony resistance, while no effect on MIT/CMIT susceptibility was observed (Fig. 3A and B). This demonstrates that the *ars* operon is functional but on its own not involved in the susceptibility to MIT/CMIT. Therefore, we assumed that the MIT/CMIT resistance determinant was located elsewhere on the plasmid.

Next, we looked for other genes that could theoretically be involved in MIT/CMIT detoxification. The plasmid contains an operon encoding a glutathione transferase (*gstA*) and a hydroxyacylglutathione hydrolase (*gloB*), here referred to as the “glutathione operon.” Whole genome sequencing of strain PHS-894 revealed more than 10 putative *gstA* homologs and one putative chromosomal *gloB* gene. The plasmid-encoded *gstA* shares 57% pairwise sequence identity with the most similar chromosomal *gstA* homolog, while the plasmid-encoded *gloB* shares 40% pairwise identity with the chromosomal *gloB* gene. Glutathione transferases are known to detoxify xenobiotic substrates (39), while isothiazolone biocides are known to react with glutathione (40). We hypothesized that these enzymes may be able to catalyze a reaction of MIT/CMIT with glutathione, which prevents MIT/CMIT from inactivating essential enzymes. Therefore, we cloned this operon into pUC19 and tested its effect on the susceptibility of recipient *E. coli* 10*β* to antimony and MIT/CMIT. The expression of the glutathione operon resulted in an approximately eightfold increase in the MIC for MIT/CMIT but had no effect on antimony susceptibility (Fig. 3A and C through F).

Finally, to confirm the role of glutathione in the detoxification of MIT/CMIT, we transferred the glutathione operon into an *E. coli* strain lacking functional glutathione biosynthesis (Δ*gshA*, KEIO collection). Such strains are known to be deficient in glutathione and exhibit a higher sensitivity to metal ions and oxidative stress (41, 42). As expected, the presence of the glutathione operon did not reduce the susceptibility to MIT/CMIT in diffusion tests or according to the measured MIC of the glutathione-deficient strain (Fig. 3D). This confirms the involvement of glutathione in the detoxification of MIT/CMIT by the genes in the glutathione operon in *Escherichia coli*.

Benzisothiazolinone (BIT) is another widely used biocide related to MIT/CMIT. It has the same functional group and mechanism of action as MIT/CMIT. Therefore, cross-resistance to both biocides could be possible. However, in contrast to MIT/CMIT, BIT contains a benzene ring, resulting in a structurally larger molecule. The glutathione operon did not influence the sensitivity to BIT according to both the MIC and the diffusion assays (Fig. 3D), indicating that the mechanism is specific to MIT/CMIT. Interestingly, the susceptibility to BIT was higher in the glutathione-deficient strain (Δ*gshA*, Fig. 3D through F), both in cells carrying the empty vector pUC19 and in cells expressing the *gstA-gloB* operon.

## DISCUSSION

In this study, we describe a reduction in susceptibility to the isothiazolinone biocide MIT/CMIT mediated by a plasmid-encoded detoxification system. MIT/CMIT targets multiple essential enzymes, so that a single mutation in one of these enzymes cannot lead to bacterial resistance. Nevertheless, adaptation to MIT/CMIT (two- to fourfold increase of the MIC) has been described in *Enterobacter gergoviae* (max. MIC 6–12 mg/L) (43), the *Burkholderia cepacia* complex (max. 4 mg/L) (44), and *Pseudomonas aeruginosa* (max. 4–8 mg/L) (45, 46). Indeed, single mechanisms could not explain the observed changes in these cases. Instead, susceptibility was reduced by complex adaptations involving multiple proteins and processes. Interestingly, while an ABC transporter appeared to be involved in efflux in *P. aeruginosa* (46), efflux did not seem to play a role in *E. gergoviae* strains with reduced MIT/CMIT susceptibility (43). In *P. aeruginosa* adapted to MIT/CMIT, three enzymes involved in sulfur metabolism were found to be differentially expressed (disulfide isomerase/thiol-disulfide oxidase [upregulated], Acyl-CoA thioesterase II [upregulated], glutathione S-transferase [downregulated]) (46). However, no single enzyme was responsible for the acquired reduction in susceptibility in this case as well.

In contrast, the mechanism described here is based on the expression of one operon containing two genes, which catalyze the detoxification of MIT/CMIT in the presence of glutathione. More studies are needed to elucidate the role and mechanism of each gene in reducing susceptibility to MIT/CMIT. It would be interesting to know whether these enzymes have an affinity to the biocide by chance, for example, due to peculiarities in structure or function, or whether they adapted to detoxify MIT/CMIT under selection pressure due to the presence of the biocide in the environment. Importantly, wild-type bacteria, such as *E. coli* MG1655, as well as *K. oxytoca* ATCC 13182 and *K. pneumoniae* DSM 12082, have MIT/CMIT MIC values of 2 mg/L (Table 4), substantially below the limit of 15 mg/L in consumer products or 100 mg/L in fuel. The wide application of MIT/CMIT, especially as a preservative for fuel, can lead to the significant exposure of bacterial populations to this biocide, e.g., inside storage tanks or after leakage. Hence, environments in which bacteria are exposed to MIT/CMIT levels that are selective for resistance mechanisms may exist. In cases where the effective concentration of an antimicrobial becomes more and more diluted in the environment (for example in soil), even a small reduction of susceptibility can lead to a significant selective advantage (47–49). This selective advantage could promote the dissemination and further evolution of detoxification mechanisms. Interestingly, the isothiazolinone biocide BIT appears to be detoxified by glutathione, but not by the *gstA-gloB* operon located on the plasmid, possibly due to substrate specificity of the enzymes involved (Fig. 3D through F). This suggests that additional glutathione-dependent mechanisms for BIT inactivation may exist in wild-type *E. coli* K12.

The localization of genes detoxifying MIT/CMIT on a mobile genetic element facilitates their spread in the environment. We even showed that the transfer of the plasmid between different bacterial species is possible. The co-localization of MIT/CMIT detoxification genes with genes encoding an efflux pump results in the co-selection of both detoxification systems under selective pressure (14). Given the right selective conditions in the environment, the spread and selection of this plasmid seem plausible.

Despite difficulties to clearly define resistance and tolerance against biocides, in this case it seems appropriate to describe this phenomenon as resistance, because the genetically encoded mechanism is stable and allows the bacteria to survive higher concentrations of the biocide, even at the concentrations used in the soap sample they were isolated from. Our findings highlight important side effects of biocide usage: the emergence, evolution, and spread of resistance mechanisms. Our results show that the spread and co-selection of a biocide resistance determinant, together with other resistance mechanisms, is possible. It is alarming that a detoxification system dedicated to MIT/CMIT has emerged and allowed bacteria to contaminate a cleaning product. The survival of an opportunistic pathogen, such as *Klebsiella*, inside soap can lead to the introduction of the pathogen into (micro-)wounds during hand washing or to the unintended spread of the bacteria. The spread of bacteria by cleaning products can potentially lead to infections, especially in immunocompromised persons, after the transmission of the pathogen from contaminated hands to more sensitive regions like mucous membranes (50, 51). Therefore, such contaminated cleaning products pose a significant threat to consumer health and safety.

## Data Availability

Plasmid sequence data are available at ENA under the project accession number PRJEB89844 or as annotated text files in Genbank format (see supporting material).

## SUPPLEMENTAL MATERIAL

Text S1 aem.01970-25-s0001.txt Annotated sequence of the 80-kbp plasmid isolated from *Klebsiella* in soap, presented in GenBank format.

Text S2 aem.01970-25-s0002.txt Annotated sequence of the *gstA-gloB* operon cloned into pUC19, presented in GenBank format.

Text S3 aem.01970-25-s0003.txt Annotated sequence of the arsenic resistance operon cloned into pUC19, presented in GenBank format.

ASM does not own the copyrights to Supplemental Material that may be linked to, or accessed through, an article. The authors have granted ASM a non-exclusive, world-wide license to publish the Supplemental Material files. Please contact the corresponding author directly for reuse.
